# Audiological outcome after stapes surgery in relation to prosthesis type

**DOI:** 10.1007/s00405-023-07822-3

**Published:** 2023-01-28

**Authors:** Vanessa Handke, Parwis Agha-Mir-Salim, Paul James, Alexander Müller

**Affiliations:** 1 Department of Otorhinolaryngology, Head, Neck, Plastic and Cosmetic Surgery, Friedrichshain Clinic, Vivantes Hearing Center (HZB), Landsberger Allee 49, 10249 Berlin, Germany; 2 Department of Otorhinolaryngology, Head and Neck Surgery and Communication Disorders, HELIOS-Clinic Berlin-Buch, Berlin, Germany

**Keywords:** Stapes surgery, Prosthesis type, Air–bone gap, Audiological outcome, Surgery time

## Abstract

**Purpose:**

Different techniques are used to fix crimp and CliP® Piston stapes prostheses to the long process of the incus (LPI). The CliP® Piston provides a stiff connection in contrast to the static bended loop of the crimp prosthesis, which imitates the physiological incudostapedial joint (ISJ) and thereby potentially leads to different hearing outcome.

**Methods:**

In a retrospective single-center study of German-speaking one hundred and ninety patients who underwent stapes surgery CliP® Piston or crimp prostheses between the years of 2016 and 2019 by the same surgeon and in the same setting. Pre- and postoperative bone- (BC) and air-conduction (AC) pure-tone thresholds, pre- and postoperative air–bone gap (ABG) for 0.5, 1, 1.5, 2, 3, 4 kHz and the surgery time were examined.

**Results:**

The postoperative bone conduction thresholds were significantly lower in the frequencies between 0.5 and 3 kHz and the mean ABG was < 10 dB in most cases independent of the prosthesis used. Crimp prosthesis showed a significantly better closure of the ABG at 0.5 kHz.

**Conclusions:**

The audiological outcome after stapes surgery is dependent on the type of prosthesis used, as reflected by the frequency-specific air–bone gap. The better ABG closure with the crimp prosthesis might be the result of the connection to the LPI imitating the physiological ISJ. The crimp prosthesis may be the better choice if use of hearing aids is expected postoperatively.

## Introduction

Stapedectomy was first performed by Frederick L. Jack at the end of the nineteenth century. The technique was rediscovered by John Shea in 1956 [1], who fixed the first Teflon implant between the long process of the incus (LPI) and the oval window (OW) [2, 3]. Schuknecht was the first to fix an implant by way of crimping an intraoperatively created steel wire/fat prosthesis to the LPI [3]. Since then, a great variety of similar prostheses using different materials and fixation methods have been developed. Today, titanium CliP® Piston or crimp prostheses are most widely used, and other materials, such as platinum wire/Teflon implants less so. In the past 20 years, studies have analysed the piston diameter [4], the method for stapedotomy [5], cement fixed crimpers [6], or the different clip types [7–11]. The few studies that have compared CliP® Piston and crimp prostheses showed no significant difference in the audiological outcome [8, 9]. CliP® Piston prostheses are fixed to the LPI dynamically and are stiff due to spring tension. Crimp prostheses are fixed statically by bending a static loop around the LPI, which provides mobility similar to the physiological ISJ, which can lead to differences in the hearing outcome [12]. It is possible that the different fixation methods result in different surgical time, because whereas the CliP® Piston is pushed onto the LPI, with crimping, the surgeon must change instrument to bend the loop using, for example, crimp forceps. Conversely only one previous study compared a nitinol CliP® Piston (*n* = 9) and platinum Teflon wire crimps (*n* = 10) prostheses performed by the same surgeon. This study did not show any differences in outcome; however, this may be due to the small number of patients included [11].

The current retrospective study was designed to compare the surgery time and the audiological outcome using the same titanium crimp and CliP® Piston prosthesis type, and with the novel aspect that the 190 included surgeries were performed by a single surgeon in an identical clinical setting. The air–bone gap (ABG) and postoperative changes in the air- and bone-conduction thresholds within the speech frequency specific changes were analysed.

## Methods

### Patients and material

**Fig. 1:**
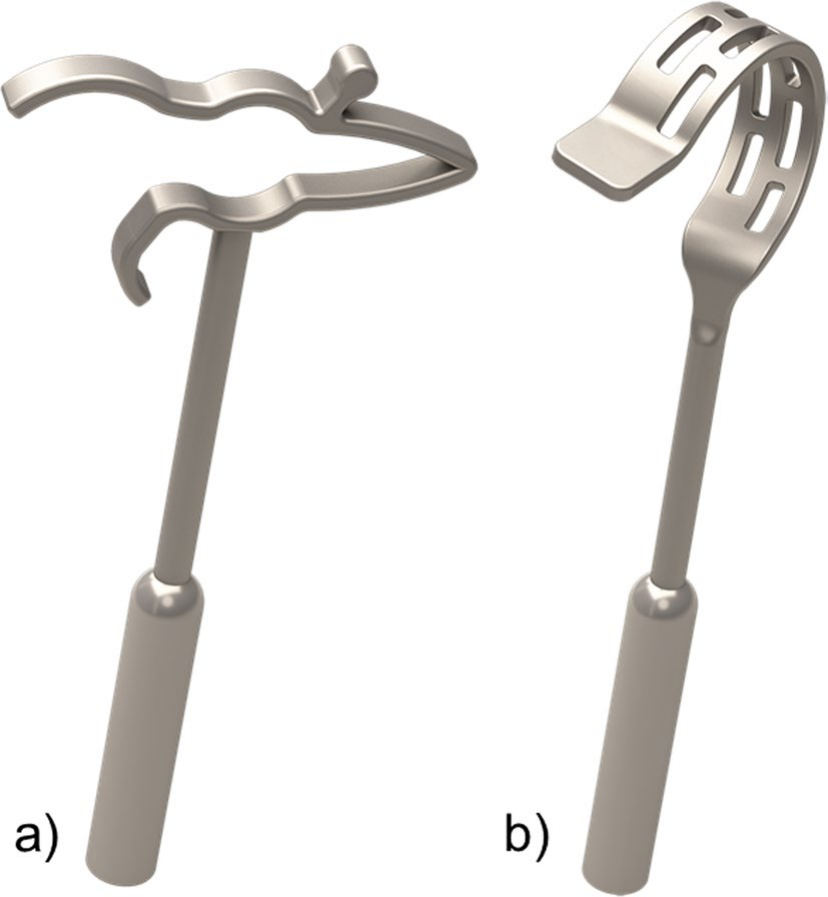
Different types of stapes prostheses were used for stapes surgery, **a**) CliP® (CliP Piston àWengen, Heinz Kurz GmbH), **b**) crimp (MatriX Stapes Prosthesis, Heinz Kurz GmbH)

In a retrospective single-center study between January 2016 and December 2019 all patients (*n* = 225) with stapes surgery were reviewed. All cases with different surgeons or revision surgeries were excluded (*n* = 35). The remaining 190 patients underwent surgery by the same surgeon (77 male,113 female; 95 left, 95 right ears; mean age 46.1 years, range 18–93). All had a mixed or conductive hearing loss and otosclerosis was confirmed intraoperatively. A CliP® Piston prosthesis was used in 112 cases and the crimp type in 78 cases (see Fig. 1). The surgeon chose intraoperatively the type of prosthesis dependent on the length and shape of the LPI. In cases of a short LPI reaching only the edge of the oval niche or if the LPI shape is curved, a CliP® Piston prosthesis can come into contact with the edge of the footplate perforation. In these cases a crimp prothesis was used and the loop tightening according to the intraoperative situation adapted.

The audiological data used in this study are based on a maximum follow-up time of 3 weeks.

### Surgery and prostheses

A microscope was used in all cases. The surgery was performed under general anesthesia. After additional infiltration of 3 ml xylocaine 2% with adrenaline 1/250000, an endaural incision was completed. After elevating a short tympanomeatal flap the tympanon was opened while protecting the chorda tympani. The lateral attic wall was curettized until visualisation of the oval window niche, the pyramid process and the facial nerve canal. After checking the ossicular chain and especially the stapes mobility, otosclerotic areas in the oval niche were examined to confirm the diagnosis. Following a footplate perforation of 0.6 mm using the Fisch perforator, the stapes superstructure was removed. The prosthesis length was determined and the prosthesis introduced using a 0.4 mm piston diameter. Clips were pushed over the LPI with a 90° hook, crimps were fixed with an alligator and/or MacGee forceps. The oval window niche was sealed with tissue graft and the restored mobility of the chain verified. Finally, the tympanomeatal flap was repositioned, covered with gelfoam, the endaural incision closed with subcutaneous sutures and the ear was packed.

### Measurements and data-processing

The surgery time in minutes was recorded between incision and suture. Audiological measurements were performed in a sound-proofed room [13] and were collected as a part of clinical routine for pre- and postoperatively diagnostic. Air- and bone-conduction pure-tone thresholds in dB HL (hearing level) were measured at frequencies of 0.5, 1, 1.5, 2, 3 and 4 kHz prior to surgery and between 2 and 3 week post-operatively. The mean air-conduction (AC) and bone-conduction (BC) pure tone average were calculated (4PTA_AC and 4PTA_BC, [0.5, 1, 2 and 4 kHz]). Furthermore, air–bone gap was calculated as the differences between air-conduction and bone-conduction thresholds separately for each measured frequency. According to the Committee on Hearing and Equilibrium of the American Academy of Otolaryngology-Head and Neck Surgery, a postoperative ABG ≤ 10 dB is an (audiometrical) criterion for successful otosclerosis surgery [14, 15].

### Statistical analysis

All statistical analyses were performed with IBM® SPSS® 25 software (IBM Corp, Armonk, NY). A confidence level of 95% and above (*p* < 0.05) was considered significant. For the CliP® Piston and crimp prosthesis types, the surgery-time and the postoperative air–bone-gap differences were compared by an independent samples Student’s *t* test. The pre- and postoperative bone-conduction pure-tone thresholds were analysed with a paired Student’s *t* test. Furthermore, descriptive frequency statistics were applied for differences in surgery time. In addition, the difference between preoperative 4PTA_AC and the postoperative 4PTA_AC was calculated and subsequently plotted in relation to the preoperative 4PTA_BC minus postoperative 4PTA_BC difference. Box plots were used to demonstrate the scattering of pre- and postoperative air–bone gap through their quartiles separately for each measured frequency.

## Results

### Surgery time

**Fig. 2:**
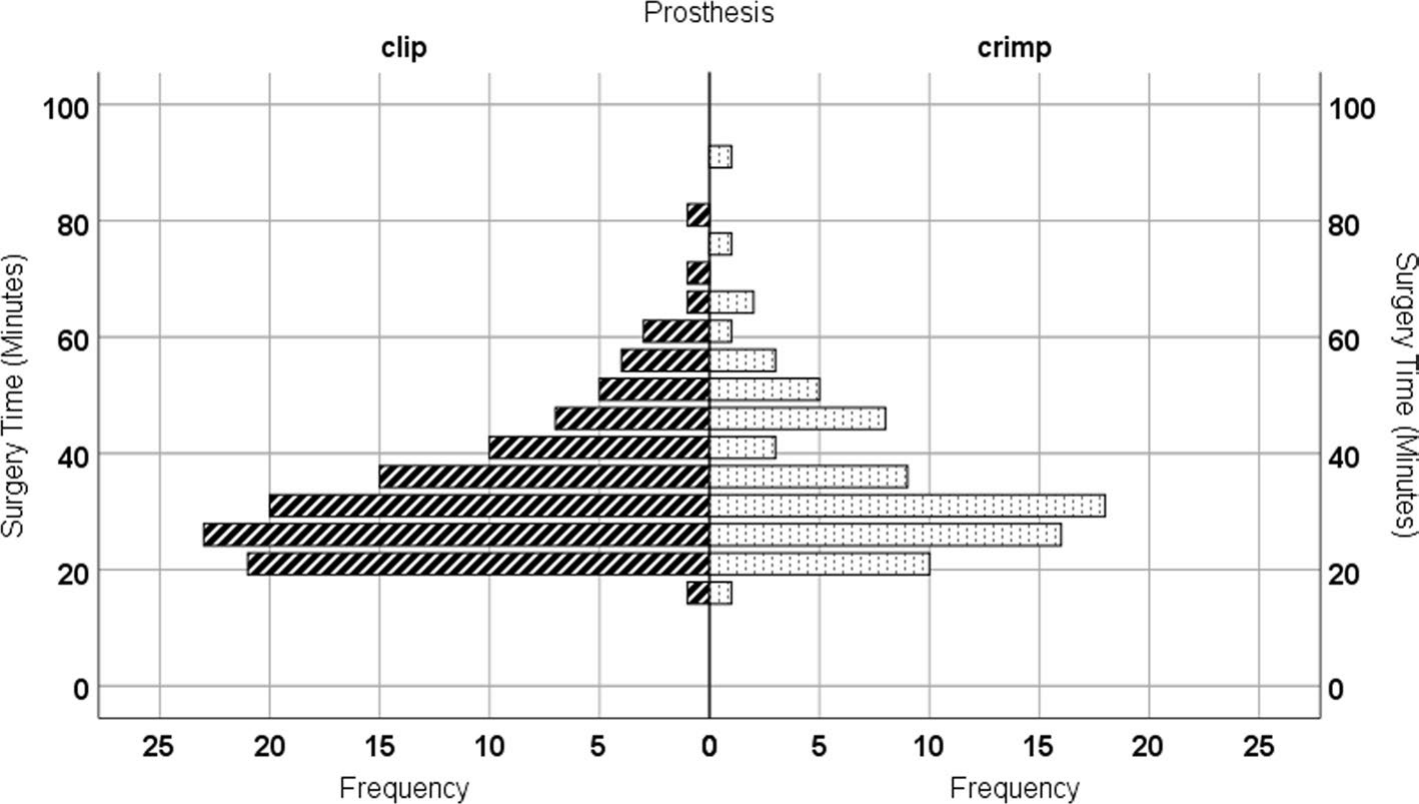
Frequencies of surgery time for two different prostheses: CliP® Piston (*n* = 112) and crimp (*n* = 78)

The mean surgery time using the CliP® Piston was 33.9 min (SD = 12.4) and was not significantly different from surgery time with the crimp prosthesis (*M* = 36.1, SD = 13.7, *p* = 0.249), see Fig. 2.

### Preoperative air–bone gap

**Fig. 3:**
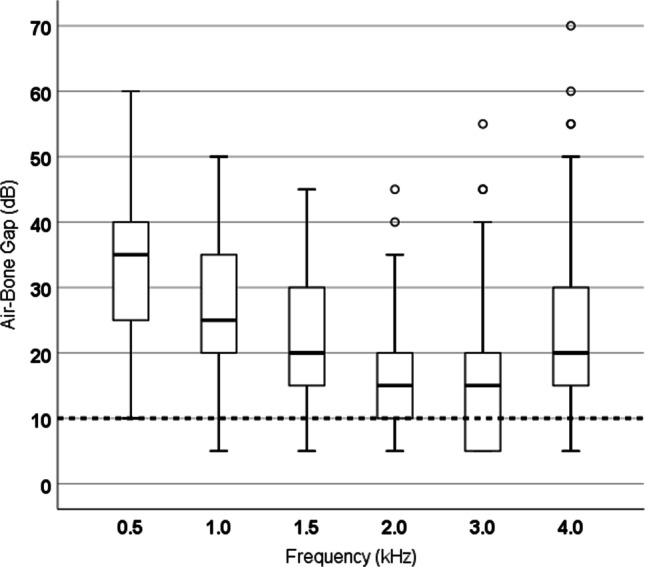
Preoperative air–bone gap (ABG) separately for each measure frequency. The dashed line marks the criterion for the best result after otosclerosis surgery (ABG ≤ 10 dB)

Figure 3 illustrates the preoperative air–bone gap for all cases at the six test frequencies. The median ABG is approximately 35 dB at 0.5 kHz, 25 dB at 1 kHz, 15 dB at 2–3 kHz and 20 dB at 4 kHz.

### Air- and bone-conduction thresholds

**Fig. 4:**
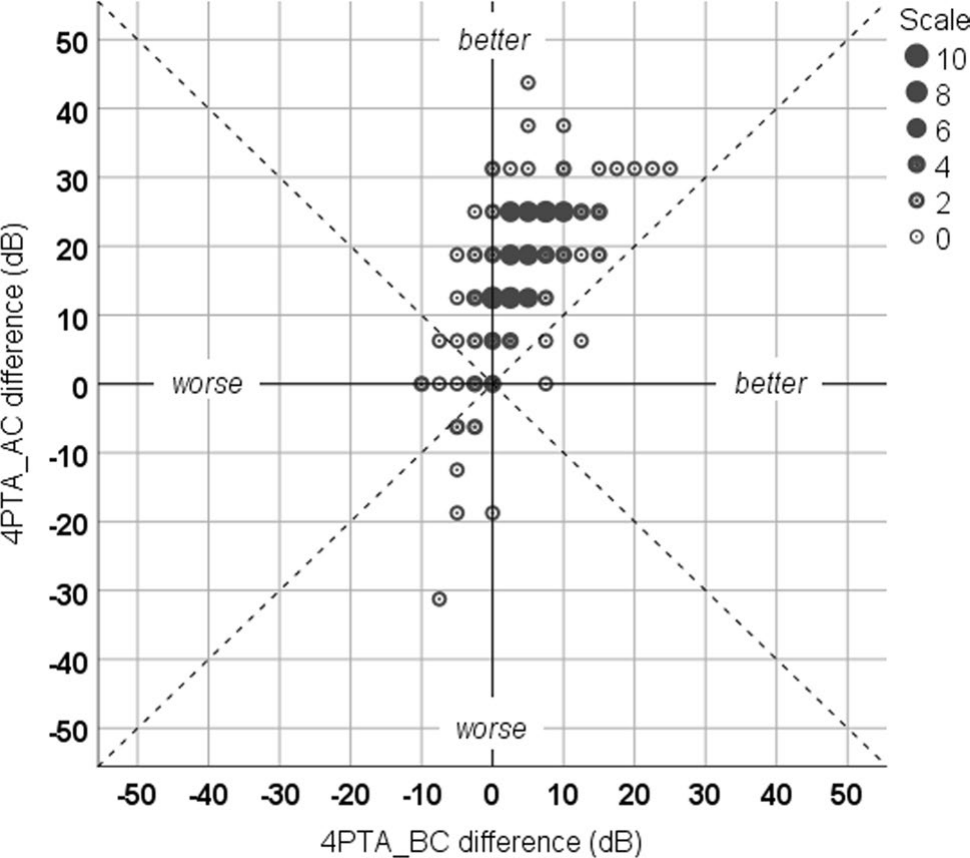
Preoperative minus postoperative 4PTA_AC difference as function of the preoperative minus postoperative 4PTA_BC difference. Each data point represent number of patients with corresponding values (see scale). PTA indicates pure tone average (0.5, 1, 2, 4 kHz); AC air conduction; BC bone conduction

Table 1 shows significant reduction (*p* < 0.001) of bone-conduction thresholds measured at frequencies of 0.5, 1, 1.5, 2 and 3 kHz on following a maximum time interval of 3 weeks after stapes plastic surgery. The mean difference of pre- and postoperative BC thresholds improve by 3.4 dB (SD = 7.2) at 0.5 kHz, 5.5 dB (SD = 7.9) at 2 kHz and 2.9 dB (SD = 9.5) at 3 kHz. Only at 4 kHz is a moderate but significant increase in the BC thresholds (*M* = 1.7, SD = 9.8, *p* < 0.05). Figure 4 shows the individual preoperative minus postoperative 4PTA_AC difference as a function of the preoperative minus postoperative 4PTA_BC difference. Mean air and bone conduction improved in approximately 2/3 of patients (see upper right quadrant). In most of these cases (datapoints over the dashed line), the average air–bone gap has also decreased. After stapes surgery the pure-tone average for bone-conduction shift (worse) was maximal 10 dB (see upper left and lower left quadrant).

**Table 1:** Statistical comparison (paired Student’s *t* test) of the change in bone conduction (BC) thresholds approx. 2–3 weeks postoperatively in patients after stapes plastic surgery: frequency (*f*), number of cases (*n*), mean differences (*M*) and standard deviation (SD) in bone conduction and *p* value

BC @ *f* (kHz)	*n*	*M* (dB)	SD (dB)	*p*
0.5	158	3.4	7.2	< 0.001
1.0	158	5.0	7.5	< 0.001
1.5	155	5.1	7.4	< 0.001
2.0	152	5.5	7.9	< 0.001
3.0	152	2.9	9.5	< 0.001
4.0	149	− 1.7	9.8	0.029

### Postoperative air–bone gap in relation to CliP® piston or crimp prosthesis

**Fig. 5:**
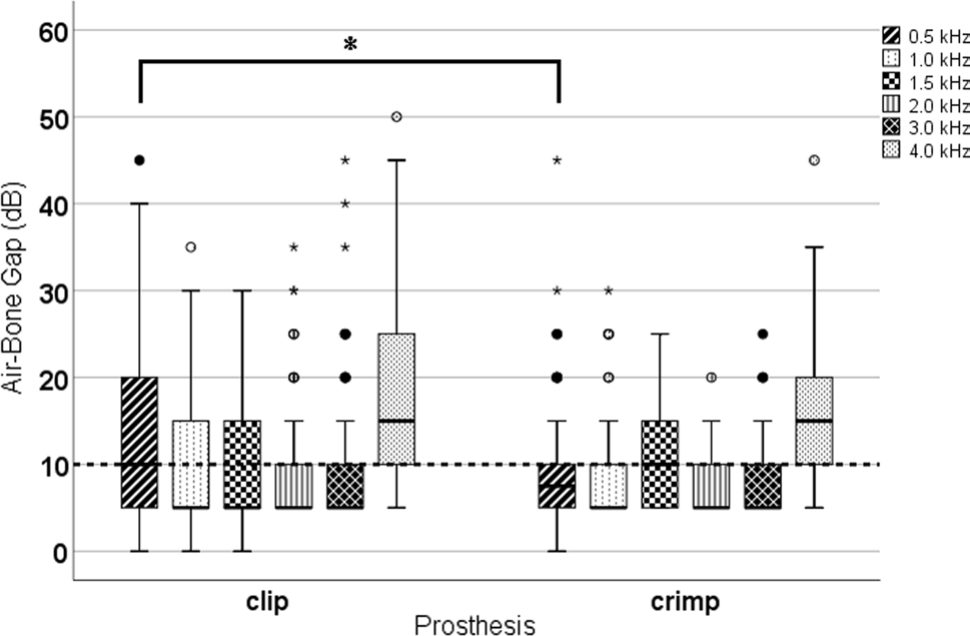
Comparison of postoperative air–bone gap for each measure frequency with CliP® Piston and crimp prosthesis. The dashed line marks the criterion for the best result in otosclerosis surgery (≤ 10 dB). Significant differences are indicated by *(*p* < 0.05)

In Fig. 5, the postoperative ABG at each frequency with CliP® Piston or crimp prosthesis was analysed. The dashed line marks the criterion for the target result in otosclerosis surgery (≤ 10 dB). A significant difference in ABG was only seen at 0.5 kHz (Table 2).

**Table 2:** Statistical comparison (independent samples Student's *t* test) of the postoperative air-bone-gap (ABG) differences between CliP® Piston or crimp prosthesis: frequency (*f*), number of cases (*n*), mean differences (*M*) and standard deviation (SD) in ABG and *p* value

ABG @ *f* (kHz)	*n* (CliP®/crimp)	*M* (dB)	SD (dB)	*p*
0.5	96/62	3.3	1.4	0.024
1.0	95/63	1.7	1.1	0.123
1.5	95/60	0.3	1.0	0.727
2.0	94/60	0.9	0.9	0.318
3.0	92/61	1.3	1.0	0.211
4.0	92/60	0.3	1.7	0.824

## Discussion

The hearing outcome after stapesplasty is determined by the bone- and air-conduction thresholds and the ABG closure. With otosclerosis most of the patients show a mixed hearing loss consisting of a conductive and sensorineural component. The difference between bone-conduction and air-conduction thresholds are defined as the air–bone gap. This parameter depends directly on both thresholds. Clinically, such a hearing loss is seen in cases of chronic middle ear disease, inner ear anomalies, and especially in otosclerosis [16].

In otosclerosis a bone conduction depression at 2 kHz is well-known and was first described by Raymond Carhart in 1950. This effect is attributed to a higher impedance at the oval window niche, which is consistent with the typical intraoperative findings. According to the literature, the frequency of occurrence of otosclerosis is between 31% and 80%. A postoperative improvement of the bone conduction threshold after stapesplasty is a well-known effect and, although most commonly observed at the Carhart’s notch, can also occur at higher and lower frequencies [17–19]. The importance of low frequencies in particular for adequate speech perception in noisy situations has been demonstrated in the literature following hybrid cochlear implantation (electro-acoustic stimulation) [20].

Our results were derived from a standardized procedure performed by one surgeon using two different prostheses. For both types we analysed the BC and the ABG at 0.5, 1, 1.5, 2, 3 and 4 kHz. In our study the BC significantly improved in all frequencies and the ABG was reduced in most of cases regardless of the prosthesis used. These positive effects are commonly observed following successful stapes surgery [21, 22].

Reduction of BC thresholds and absence of the Carhart’s notch (CN) are positive factors for postoperative hearing performance [23].

Furthermore, our results showed a significant BC improvement in all frequencies and surprisingly a significant better ABG closure at 0.5 kHz for the crimp prosthesis. Different factors such as prosthesis diameter and material, fixation at the LPI are possible factors influencing the BC shift and ABG closure.

Advantages and disadvantages of the different stapes prosthesis designs are frequently described in the literature. Nowadays, the most commonly used material is titanium, which has a low density, electrical and thermic conductivity, high stiffness and is well-tolerated within the middle ear [24, 25].

Several prosthesis types with different piston diameters and fixation methods at the LPI have been used in the past. It is well-known that the prosthesis diameter has no significant influence to the hearing outcome, and that a tight attachment to the LPI leads to a narrower postoperative AGB [12, 26]. As such, the method of prosthesis fixation at the LPI may play a major role. In a review of 17 papers reporting an ABG closure of 10 dB or less overall and 20 publications examining the mean ABG using different prostheses, the authors conclude that there is no difference in hearing outcome and no fixation method can be favoured [26]. Similar results were shown in a study comparing a CliP® Piston (*n* = 63) and a crimp (*n* = 63) prosthesis. Analysing mean values of the residual ABG, no significant differences could be demonstrated and both had good functional results [27]. In none of these studies was the ABG analysed separately for each frequency.

Nowadays the ossicular middle ear joints are considered as a mechanism not for lever induced sound amplification, but rather for realization of an adequate sound transmission under different pressure conditions of this system [25]. On the other hand, the incudostapedial joint plays an important role for middle ear sound transmission especially in the lower frequencies. An experimental fixation of the incudostapedial joint and footplate showed a decrease of sound transmission of 10–25 dB and 20–30 dB at 1 kHz and below [28]. Similar results with 3–6 dB less sound transmission were shown in an experimental study using artificial stapes fixation by cyanoacrylate adhesive [29].

In our study the prosthesis material and piston diameter for both types were identical. In consequence, systematical differences in hearing outcome can only be related to the fixation method.

Our results show that the sound transmission is significantly better at 0.5 kHz for the crimp prosthesis, which represents a clear advantage. A CliP® Piston creates a stiff connection between the LPI and the prosthesis. A crimp type is fixed with a tight bended loop that retains mobility and better mimics the ISJ, which may account for better sound transmission in the lower frequencies.

Despite these aspects many patients postoperatively need a hearing aid with an appropriate earmold fitting. From approx. 35 dB HL in the low-frequency range, an occluding earmold with a small ventilation hole (≤ 1 mm) may be necessary. This can lead to increased moisture, cerumen buildup and also to external auditory canal infections. In addition, tight occlusion causes a significant reduction in sound quality. To avoid such problems, the smallest possible air–bone gap (≤ 10 dB of 0.5 kHz) is a desirable result of stapes surgery.

The results of our study show that this goal could be reached in more than 70% of the patients using both the CliP® Piston and crimp prosthesis. In comparison, a crimp prosthesis is significantly more efficient at transduction of the important lower frequencies. Limitations to the presented study are that it was not randomised and that audiological measurements should be conducted over a longer period to assess the long term results.

## Conclusion

In most of the cases stapes surgery leads to a hearing improvement by elevating the BC in combination with closing of the ABG between 1 and 4 kHz. Our results are consistent with the pertinent literature. Comparing the CliP® Piston and crimp prostheses the crimp showed a significant better ABG closure at 0.5 kHz. In future a long term prospective study is planned to investigate whether this has an effect on speech perception. Low frequencies are known to be important for speech perception in noise. Therefore, an improvement in low frequency thresholds may considered a clear advantage for the audiological outcome, especially in cases, where postoperatively hearing aids are indicated.

## Data Availability

The datasets generated for this study are available on request to the corresponding author.
